# Can Interferon-Gamma or Interferon-Gamma-Induced-Protein-10 Differentiate Tuberculosis Infection and Disease in Children of High Endemic Areas?

**DOI:** 10.1371/journal.pone.0023733

**Published:** 2011-09-23

**Authors:** Mohammed Ahmed Yassin, Roberta Petrucci, Kefyalew Taye Garie, Gregory Harper, Isabel Arbide, Melkamsew Aschalew, Yared Merid, Zelalem Kebede, Amin Ahmed Bawazir, Nabil Mohamed Abuamer, Luis Eduardo Cuevas

**Affiliations:** 1 Liverpool School of Tropical Medicine, Liverpool, United Kingdom; 2 Faculty of Medicine, University of Hawassa, Awassa, Ethiopia; 3 Bushullo Major Health Centre, Awassa, Ethiopia; 4 Southern Region Health Bureau, Awassa, Ethiopia; National Institute of Allergy and Infectious Disease, United States of America

## Abstract

**Background:**

Diagnosis of childhood tuberculosis (TB) is difficult in high TB burden settings. Interferon-gamma-induced protein 10 (IP10) has been suggested as a marker of TB infection and disease, but its ability to differentiate the two conditions remains uncertain.

**Objectives:**

To describe Interferon-gamma (INFγ) and IP10 expression in children with TB infection and disease and controls to assess their potential to differentiate latent and active TB.

**Methods:**

This was a cross sectional study of 322 1–15 years old children with symptoms of TB (28 *confirmed*, 136 *probable* and 131 *unlikely* TB), 335 children in contact with adults with pulmonary TB and 156 community controls in Southern Ethiopia. The Tuberculin Skin Test (TST) and Quantiferon-In-Tube (QFT-IT) were performed. INFγ and IP10 were measured in plasma supernatants.

**Results and Interpretation:**

Children with *confirmed* and *probable* TB and *contacts* were more likely to have TST+ (78.6%, 59.3% and 54.1%, respectively) than children with *unlikely* TB (28.7%) and *controls* (12.8%) (p<0.001). Children with *confirmed* TB (59.3%) and *contacts* (44.7%) were more likely to have INFγ+ than children with *probable* (37.6%) or *unlikely* TB (28.1%) and *controls* (13.1%) (p<0.001). IP10 concentrations were higher in INFγ+ children independently of TST (p<0.001). There was no difference between IP10 concentrations of children with *confirmed* TB and *contacts* (p = 0.8) and children with and without HIV (p>0.1).

INFγ and IP10 can identify children with TB infection and disease, but cannot differentiate between the two conditions. HIV status did not affect the expression of IP10.

## Introduction

One of the most significant barriers for the appropriate management of Tuberculosis (TB) is the lack of suitable diagnostic tests. This problem is especially critical in settings where facilities are limited and other infections with overlapping clinical presentation are highly prevalent. Childhood TB presentation differs from adult TB as children experience more extra-pulmonary TB (EPTB) and lung involvement is frequently disseminated and without cavitations. Young children are unable to expectorate sputum and the specimens collected, such as gastric aspirates, are often paucibacillary and sometimes contain test inhibitors. Most diagnostics for TB thus perform poorly in children [1], [2] and new tests or markers to diagnose TB in children are needed.

Interferon-gamma (INFγ) release assays (IGRAs) were developed in the last decade and are reported to have comparable sensitivity and higher specificity than the Tuberculin Skin Test (TST) to identify latent TB infections (LTBI). Their performance however is less reliable in young children and in patients co-infected with the Human Immunodeficiency Virus (HIV) [3], [4], [5]. These assays are frequently used by clinicians to support a diagnosis of symptomatic TB [3], [6], [7], even though they cannot distinguish between LTBI and symptomatic disease. IGRAS are also increasingly used in high TB incidence settings, despite the limited information of their performance in these locations [8], [9], [10].

INFγ-induced protein 10 (IP10, also known as Chemokine (C-X-C motif) ligand 10 or CXCL10) is another biomarker recently reported to identify LTBI, which, when combined with IGRAS is said to increase the sensitivity of the assays [10]. IP10 expression is putatively less affected by HIV [11] and young age [12], [13] and it is said to have potential to differentiate between LTBI and symptomatic infections [12], [14].

We have thus conducted a study in Southern Ethiopia to describe INFγ and IP10 concentrations of children presenting with symptoms compatible with TB, asymptomatic children in contact with adults with smear-positive pulmonary TB (PTB) and asymptomatic community controls to explore whether INFγ or IP10 can distinguish between symptomatic and asymptomatic infections in a high TB burden setting and assess whether these markers could be used to support the diagnosis of children with symptoms of TB.

## Materials and Methods

This was a cross sectional study of 1 to 15 year old children with symptoms suggestive of TB, children in contact with adults with smear-positive PTB and community controls without known contact with TB. The study was based in Awassa, in the Southern Region of Ethiopia, which has a population of over 15 million.

Children with symptoms of TB (called *symptomatics*) were enrolled consecutively at three health service providers (Awassa Health Centre, Bushullo Major Health Centre and University of Hawassa referral hospital). These included consecutive children with cough or fever for more than 2 weeks, night sweats, anorexia, weight loss, failure to thrive or non-specific symptoms in the presence of a history of contact with an adult with PTB. Children were invited to participate at the time of presentation to the clinic. All children underwent a clinical examination, chest X-rays, TST, three sputum or gastric lavage examinations and fine needle aspiration if enlarged lymph nodes were detected. All specimens were examined using light smear microscopy and culture on Lowenstein Jensen media and children were classified as having *confirmed* (positive smear microscopy or culture), *probable* (negative smear microscopy and culture, clinical and radiological findings consistent with TB, no improvement after a full course of antibiotics and the clinician initiating anti-TB treatment) or *unlikely* TB (children with alternative diagnosis or clinical improvement after a course of broad spectrum antibiotics).

In addition, *contact* children 1–15 year old residing with adults with smear-positive PTB (called *contacts*) were enrolled by identifying adults attending the same study centres with a history of cough for more than 2 weeks duration who had sputum smear-positive PTB. Once a smear-positive adult had been identified, study investigators inquired whether they had children at home and visited the households if the family resided within a 20 km radius from Awassa. All children living under a single roof who shared meal times with the adult were invited to participate after obtaining informed parental consent, unless they had previously received treatment for TB. All children who shared meals at home with the index cases were included, regardless of the duration of contact.

Community *controls* were defined as children residing in the community who did not have a known contact with adults with PTB. *Controls* were selected from Awassa and surrounding *kebeles*, which are the smallest administrative units of Ethiopia and comprise at least five hundred families or 3,500 to 5,000 inhabitants. A list of all villages and *kebeles* located within a 20 Km radius from Awassa was constructed. Controls were enrolled from villages adjacent to the villages where contacts resided. We enrolled more controls from rural villages than from urban areas as the risk of TB transmission from casual contact could be higher among urban residents. Households within a village were selected by spinning a pen in a street somewhere between the centre and the edge of the village and children were enrolled after obtaining informed parental consent. Households were excluded if the parent indicated the family had been in contact with an adult with cough for more than 2 weeks duration in the previous 2 years or if a family member had received treatment for TB. All children were applied a TST using 2 units of Purified Protein Derivate (PPD, RT 23, Statens Serum Institute, Copenhagen, Denmark) using the Mantoux method and indurations were measured using the palpation method 48 to 72 hours later. TST results were graded as negative (<5 mm), intermediate (≥5 and <10 mm) and positive (≥10 mm). Any child with signs and symptoms suggestive of TB was investigated for disease activity and treated. *Contact* children <6 years old were offered INH prophylaxis for 6 months independently of their TST result, following Ethiopian TB Control Programme guidelines.

INFγ and IP10 concentrations were assayed using commercially available Enzyme Linked Immunoassay (ELISA) kits. INFγ was measured using the QuantiFERON-TB Gold In-Tube (QFT-IT) test (Cellestis, Victoria, Australia). Blood samples for QFT-IT were collected in 3 tubes, one containing the *Mycobacterium tuberculosis* ESAT-6, CFP10 and TB7-7 antigens; one with non-specific mytogen (phytohemoagglutinin) to serve as positive control and one blank tube (nil) to serve as negative control. The three tubes were incubated at 37° for 16–24 hours. Supernatant plasma was harvested after centrifugation to separate the blood cells and stored at −70°C. INFγ was measured using the manufacturer's ELISA in a Bio-Rad Plate reader (Model n 550) and read at 450 nm. IGRAs were classified as positive, negative or indeterminate using the manufacturer's software. IP10 concentrations were measured in the same supernatant using a Human IP10 ELISA Construction Kit (Antigenix America Inc, New York, NY). HIV infections were established using two blood-based ELISA methods.

Means and standard deviations (SD) or medians and inter-quartile ranges (IQR) were used to describe continuous variables. Parametric tests and non parametric tests were used to compare means and medians. The proportion of children in each study category with positive/negative tests compared using Chi-Square and Fisher's exact tests (if expected values were less than<5) and the agreement between TST and INFγ was described using Kappa statistics. IP10 concentrations were described using boxplots stratified by TST and INFγ concordance. The sample size calculations took into account the feasibility to visit about 200 households (of index cases) within the available time and resources and that each household had an average of two children <5 years old and it was expected that 200 children with signs and sypmtoms of TB would attend the health services. It was expected that 50% of the participants would be TST positive and 70% and 30%, of TST-positive children would be INF-γ positive and negative, respectively. It was also assumed that 30% and 70% of TST-negative children would be INF-γ positive and negative, respectively. This sample size would allow estimating the proportion of children with positive and negative INF-γ results by study group with a precision of ±7%. Sample size calculation for the comparison of quantitative data (i.e. differences in IP-10 concentrations by study group) would have required prior information of the dispersion of IP-10 concentrations to estimate standard deviations or medians. As this information was not available, this study should be considered “exploratory”, as it might not have enough power for the differences reported. The ability of IP10 to discriminate between children with and without TB infection or disease was evaluated using Receiver Operating Characteristic (ROC) curves using Prism 5 software (GraphPad PRISM, version 5). ROC curves were constructed with children with *confirmed* TB and controls and *contact* children with TST+/INFγ+ and *controls*. Only *controls* with TST-/INFγ- results were included in the ROC analysis to select children unlikely to have TB infection. The area under the curve (AUC) for both *confirmed* and *contact* children were compared to ascertain whether IP10 could discriminate between the two conditions. Statistical tests were considered significant if p values were <0.05.

The study protocol was approved by the Health Bureau of the Southern Region and the Research Ethics Committees of the Liverpool School of Tropical Medicine, Hawassa University and the Sciences and Technology Commission of Ethiopia. The study protocol was registered in the clinicatrials.gov clinical trials register (registration number NCT00456469)

## Results

**Figure 1:**
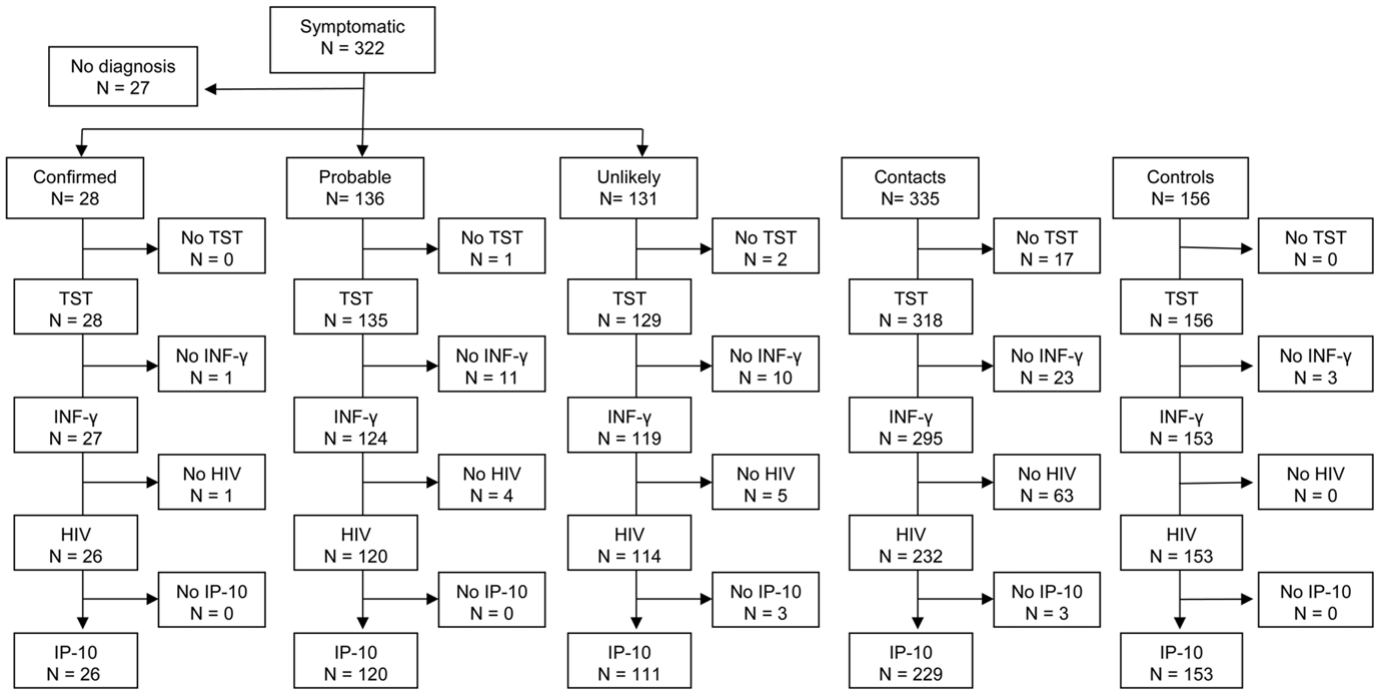
Flow chart of study participants.

Eight hundred thirteen, 322 *symptomatic*, 335 *contact* and 156 *control* children were enrolled. The flow chart of the three groups is described in Figure 1. Twenty eight (8.7%) of 322 *symptomatic* children had *confirmed*, 136 (42.2%) *probable* and 131 (40.7%) *unlikely* TB and 27 were excluded because a definitive diagnosis was not reached or the child left the hospital before completing the investigations. The 335 *contact* children were recruited from 125 adult index cases who had “scanty” (18, 5.5%), “+” (188, 57%), “++” (109, 33%) or “+++” (15, 4.5%) smear microscopy grades in their sputum smear examination. In five *contacts* the smear result of the adult was not recorded. The median contact duration was 10 hours per week (range of 1 to 41 hours). The characteristics of the participants are shown in Table 1. Children with *probable* TB were more likely to be male (67.6%), while children in the other groups had similar proportions of males and females. Children had similar median age with symptomatic and control children having a median age of 6 years and contacts having a median of 8 years. Although the proportion of children reporting to have received BCG was similar across the groups, BCG scars were found less frequently in children with *confirmed* and *probable* TB (p<0.001). HIV was positive in none of the children with *confirmed* TB, 14 (10.9%) children with *probable* and 8 (6.5%) *unlikely* TB cases, 27 (8.2%) *contacts* and 3 (1.9%) *controls* (p = 0.005). The number of residents per household was higher among children with *symptoms* of TB than among *contacts* and *controls* (means 8, 7.1 and 6.4 persons, respectively, p = 0.003). A significantly high proportion of children had lost one or both parents, with *symptomatic* and *contact* children being more likely to have lost a parent than *controls* (p = 0.01).

**Table 1:** Characteristics of the study children.

Variables	Symptomatic cases N = 295					
	*Confirmed TB* N = 28	*Probable TB* N = 136	*Unlikely TB* N = 131	Contacts N = 335	Controls N = 156	p
Male/Female (% Male)	13/15 (46.4)	92/44 (67.6)	67/64 (51.1)	168/166 (50.3)	79/77 (50.6)	
Median age (years)	8	5	7	8	6	<0.001
Range	1–15	1–15	1–15	1–15	1–15	
Received BCG	14 (63.6)	69 (63.3)	82 (70.7)	249 (74.3)	120 (76.9)	0.01
BCG scar present (%)	7 (25)	35 (25.7)	51 (38.9)	174 (51.9)	93 (59.6)	<0.001
Mean (SD) BCG scar (mm)	7.4 (3.2)	5.3 (1.8)	5.5 (2.1)	4.7 (2.4)	5.4 (2.5)	<0.001
Range	4–13	2–9	2–10	1–13	2–13	
HIV status, Pos/tested (%Pos)	0/27 (0)	14/114 (10.9)	8/116 (6.5)	27/231 (8.2)	3/153 (1.9)	0.005
Mean (SD) residents/household	8.3 (7.5)	8.9 (8.3)	7.2 (6.3)	7.1 (2.6)	6.4 (2.4)	0.002
Illiterate Father, N (%)	17 (70.8)	63 (55.8)	55 (48.7)	112 (38.6)	87 (60.4)	<0.001
Mother, N (%)	21 (84)	97 (77.6)	87 (68.5)	170 (52.3)	123 (78.8)	<0.001
Alive Father, N (%)	24 (85.7)	113 (83.1)	113 (86.3)	293 (88.3)	144 (92.3)	0.2
Mother, N (%)	25 (89.3)	124 (92.5)	128 (98.5)	325 (97.6)	156 (100)	<0.001
Both parents, N (%)	23 (82.1)	109 (81.3)	112 (80)	293 (88)	144 (92.3)	0.01

TST and INFγ results are shown in table 2. Children with *confirmed* and *probable* TB and *contacts* were more likely to have positive TST (TST+) (78.6%, 59.3% and 54.1%, respectively) than children with *unlikely* TB (28.7%) and *controls* (12.8%) (p<0.001). A high proportion of children had indeterminate INFγ tests, with children with *probable* and *unlikely* TB having a higher proportion of indeterminates than children with *confirmed* TB, *contacts* and *controls*. This problem was mostly due to failure of the positive control tube and was not associated with HIV infection (26/52 (50%) in HIV positive, compared to 256/641 (40%) in HIV-negative children, p = 0.2).

**Table 2:** TST and INFγ results and concordance among the tests by study group.

Variables	Symptomatic cases N = 295				
	*Confirmed TB* N = 28 (%)	*Probable TB* N = 136 (%)	*Unlikely TB* N = 131 (%)	Contacts N = 335 (%)	Controls N = 156 (%)
TST					
Positive	22 (78.6)	80 (59.3)	37 (28.7)	172 (54.1)	20 (12.8)
Intermediate	0	10 (7.4)	9 (7)	48 (15.1)	15 (9.6)
Negative	6 (21.4)	45 (33.3)	83 (64.3)	98 (30.8)	121 (77.6)
INFγ					
Positive	16 (59.3)	47 (37.6)	34 (28.1)	139 (44.7)	20 (13.1)
Indeterminate	5 (18.5)	47 (37.6)	44 (36.4)	52 (16.7)	25 (16.3)
Negative	6 (22.2)	31 (24.8)	43 (35.5)	120 (38.6)	108 (70.6)
TST/INFγ concordance					
TST+/INFγ+	15 (68.2)	38 (52.1)	14 (20.6)	87 (41.6)	6 (5.3)
TST+/INFγ−	3 (13.6)	15 (20.5)	10 (14.7)	39 (18.7)	10 (8.8)
TST−/INFγ+	1 (4.5)	5 (6.8)	15 (22.1)	24 (11.5)	12 (10.5)
TST−/INFγ−	3 (13.6)	15 (20.5)	29 (42.6)	59 (28.2)	86 (75.4)

Children with *confirmed* TB (59.3%) and *contacts* (44.7%) were also more likely to have positive INFγ (INFγ+) than children with *probable* (37.6%) or *unlikely* TB (28.1%) and *controls* (13.1%) (p<0.001). Twenty two (78.6%) children with *confirmed*, 73 (53.7%) with *probable* and 68 (52%) with *unlikely* TB, 209 (62.4%) *contacts* and 114 (73.1%) *controls* had paired TST and IFNγ results after exclusion of intermediate TST and indeterminate INFγ tests. Concordance between INFγ and TST varied across the groups. Children with *confirmed* and *probable* TB and *contacts* were more likely to have concordant positive TST and INFγ (15 (68.2%) of 22, 38 (52.1%) of 73 and 87 (41.6%) of 209, respectively), while children with *unlikely* TB and *controls* were more likely to have concordant negative TST and INFγ (29 (42.6%) of 68 and 86 (75.4%) of 114, respectively). Discordant TST+/INFγ− results were more frequent among children with *probable* TB (20.5%) and *contacts* (18.7%) and discordant TST−/INFγ+ results were more frequent among children with *unlikely* TB (22.1%) and *controls* (11.5%). Agreement between TST and IFNγ tests varied from 81% (k = 0.49) in children with *confirmed* TB, 73% (k = 0.40) in *probable* TB and 66% (k = 0.23) in *unlikely* TB, 70% (k = 0.39) in *contacts* and 81% (k = 0.24) in *controls*.

**Figure 2:**
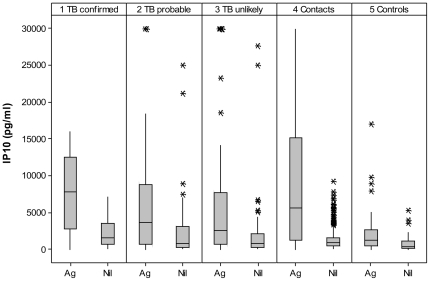
IP10 (pg/ml) concentrations in stimulated (Ag) and non-stimulated (Nil) samples by study group. Box plots describe medians, interquantile values and range. Asterisks depict outliers.

**Figure 3:**
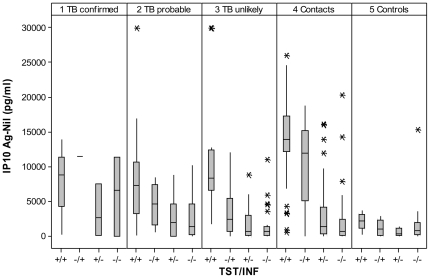
IP10 concentrations (stimulated minus non-stimulated) by TST and INFγ results and study group. Box plots describe medians, interquantile values and range. Asterisks depict outliers.

**Figure 4:**
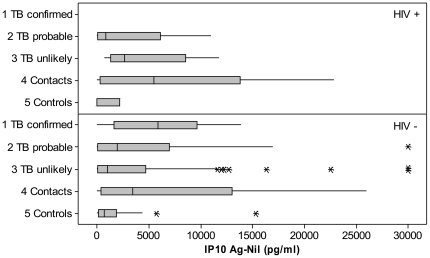
IP10 concentrations (stimulated minus non-stimulated) by HIV status and study group. Box plots describe medians, interquantile values and range. Asterisks depict outliers.

IP10 concentrations are shown in Figure 2. Stimulated concentrations were much higher than non-stimulated concentrations in all groups (p<0.001). Non-stimulated concentrations were highest in children with *confirmed* TB and lowest in *controls* (medians 1606 and 386 pg/ml). Stimulated concentrations were higher in children with *confirmed* TB and *contacts* (7812 and 5616 pg/ml, respectively) than in children with *probable* and *unlikely* TB (3680 and 2532 pg/ml, respectively, Kruskal-Wallis, p = 0.02). The difference of stimulated minus non-stimulated concentrations are shown in Table 3 by TST and INFγ results and study group. Among patients with positive TST, *symptomatic* and *contact* children had higher IP10 concentrations than *controls* (p = 0.03 and p<0.001, respectively). Among children with negative TST, children with *confirmed* TB and *contacts* had higher IP10 concentrations than *controls* (p = 0.02 and p = 0.001, respectively. There was no difference in the IP10 concentrations across study groups among children with INFγ− results, while *contact* children with INFγ+ results had the highest IP10 concentrations, followed by *symptomatic* and *control* children. Median (IQR) IP10 concentrations (stimulated minus non-stimulated) are shown in Figure 3 by TST and INFγ concordance. Children with concordant TST+/INFγ+ had higher IP10 concentrations than children with TST−/INFγ− results in all study groups. Children with *confirmed* TB had high IP10 concentrations independently of the TST or INFγ result including two of the three children who had TST−/INFγ− results. In contrast, most *controls* had low IP10 concentrations with the exception of children with TST+/INFγ+ results, who had high IP10 concentrations (p = 0.04). Children with HIV had similar IP10 concentrations than children without HIV across all study groups (p>0.1) as shown in Figure 4.

**Table 3:** Medians and interquantile ranges of IP10 concentrations (stimulated minus non-stimulated, in pg/ml) according to the TST and INFγ results by study group.

	TST			INFγ		
	Positive	Intermediate	Negative	Positive	Indeterminate	Negative
TB confirmed	5116 (2092–9624)	-	6788 (1672–11344)	8848 (4296–11414)	1672 (56–2092)	4560 (8–7496)
TB probable	2592 (64–8272)	768 (0–7424)	512 (84–3348)	7254 (3360–10548)	84 (0–604)	1288 (140–4548)
TB unlikely	2404 (326–8868)	2903 (692–8688)	674 (54–2558)	7594 (3328–12028)	548 (0–2208)	674 (120–1372)
Contacts	9304 (708–14536)	6078 (476–17632)	1486 (308–5544)	13800 (11128–17728)	348 (20–1932)	770 (196–3224)
Controls	528 (72–1392)	1124 (40–2320)	704 (116–1932)	1784 (364–2562)	180 (0–1184)	684 (144–1792)

**Figure 5:**
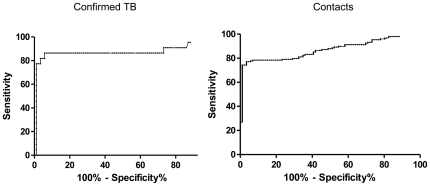
Receiver Operating Characteristic (ROC) curve of IP10 in children with *confirmed* TB and *contacts*, when compared to community *controls* with TST- and INFγ results.

IP10 concentrations of children with *confirmed* TB (N = 22, median IP10 7774 pg/ml) and *contacts* with TST+ and/or INFγ+ (N = 148, median IP10 12490 pg/ml) where compared against *controls* with TST− and INFγ− (N = 86, median IP10 704 pg/ml) to construct the ROC curves, as shown in Figure 5. The ROC curves for both *confirmed* and *contact* children had the same shape, with cut-off points for best performance being 3128 pg/ml (sensitivity 81.8%, 95%CI = 59.7–94.8, specificity 96.5% 95%CI 90.1–99.3 and area under the curve (AUC) of 0.87%, 95%CI 0.75–0.99) for *confirmed* TB and 3022 pg/ml (sensitivity 77%, 95%CI 69.4–83.5%, specificity 96.5%, 95%CI 90.14–99.3%, and an AUC of 0.87, 95%CI 0.82–0.92) for *contacts*.

## Discussion

Despite significant research efforts and technological breakthroughs to develop new diagnostics for TB [15], current diagnostic tests have lower sensitivity in children than in adults [1], [2]. New diagnostics are needed to identify children with TB and to differentiate between latent and active TB in high incidence settings with limited resources.

Despite a large body of evidence of the performance characteristics of IGRAs for the diagnosis of LTBI and the identification of individuals infected during TB outbreaks in low TB incidence settings [6], [16], [17], [18], [19], there is a small number of studies assessing the IGRAS performance and their utility in high incidence countries [20], [21], [22]. The data presented here therefore represents a rare opportunity to compare TST, INFγ and IP10 in children with different degrees of exposure to infection and certainty of diagnosis residing in a high TB burden setting.

An important difference to reports from industrialized countries was the high proportion of QFT-IT tests with indeterminate results. Other studies from Africa have reported high rates of indeterminate results [23], and the reason for this high frequency remains unexplained. Our team has conducted similar studies in Nigeria [24], Nepal [8] and Yemen, took care to re-stock tests frequently and used high altitude control tubes provided by the manufacturer. Most indeterminate results however were due to failure of the positive control and further studies are needed to explore whether this was due to a loss of test integrity or an unidentified background morbidity such as parasitic, bacterial or viral infections [25]. The interpretation of the data is also hampered by the lack of reference standards for LTBI. The data however confirms that INFγ, as TST, are more likely to be positive in children with *contact* or *confirmed* TB than in *controls*. Neither INFγ nor TST differentiate between active and latent infections and thus their diagnostic value is restricted to the confirmation of a history of infection [3], [19], [26]. Given that a number of children had discrepant INFγ/TST results, cost and logistic constrains aside, the use of both TST and INFγ would identify a higher number of children with evidence of infection than a single test alone.

This study also describes IP10 concentrations of children at different risk of infection, and how these concentrations vary with TST, INFγ and HIV. IP10 is a cytokine expressed in response to IFNγ stimulation by cell types involved in delayed-type hypersensitivity [27], including lymphocytes, monocytes, endothelial cells and fibroblasts and is a chemo-attractant to monocytes and activated Th1 lymphocytes [28], promoting selective enhancement of Th1 responses and increasing IFN-γ gene expression [29]. Recent studies have reported that IP10 expression is enhanced in individuals with active TB [30], [31] and latent infection [12], [13], [32] and that combined with INFγ could increase the sensitivity of the IGRAS [9], [12]. Although some reports have suggested that this marker could differentiate between active and latent infection [14], [33], not all studies have replicated these observations [10], [14].

IP-10 concentrations were higher among children with *confirmed* TB and *contacts*, with the highest concentrations observed in *contact* children, which is in agreement with recently recent reports from low incidence settings [12], [14]. IP10 concentrations were generally higher among children with INFγ+ results and there was no difference in the median concentrations of children with and without HIV infection. This latter characteristic could have practical applications, increasing the sensitivity of INFγ based tests in areas of high HIV prevalence.

Interestingly, non-stimulated IP10 concentrations were higher in children with *confirmed* TB, resulting in a smaller difference between stimulated and non-stimulated concentrations. Although non-specific IP10 increases have been described in acute and chronic diseases other than TB [34], [35], [36], [37], these findings suggest that IP10 expression may vary during the course of disease progression, maybe with higher expression during the primary stages of granuloma formation, which involves the recruitment of monocytes and Th1 gammadelta T-cells [38] and lower expression in the presence of progressive unchecked infections, in the presence of immunosuppression, as described for INFγ.

The ROC curve analysis however suggested that the cut offs with best test performance to differentiate children with *confirmed* TB and *controls* was similar to the cut offs to differentiate *contacts* and *controls*, with similar AUC. Thus, similar to INFγ and TST, IP10 differentiated between infected and non-infected children but is unable to differentiate between latent and active TB. Data from children residing in a high burden areas is scarce and the sensitivity of 81.8% obtained is lower than reported from Indian adults [10] in Chennai (92.5%, 95%CI: 88.6%–96.4%), which also reported a lower specificity (48%). Ruhwald et. al. [39] selected a cut off of 673 pg/ml for Danish adolescents and adults and Lighter et. al [12] selected a cut off of 732 pg/ml for USA children. Applying Ruhwald et. al., cut off to Ethiopia would increase the sensitivity to 86% and reduce the specificity to 48%, which is similar to those reported by Kabeer et al in India, suggesting that there is more agreement of test performance across settings than suggested by these reports.

This study therefore describes INFγ and IP10 expression in children at different risk of infection and disease in a country with a high incidence of TB. Children with *probable* TB had different tests results patterns than children with *confirmed* TB as the former had more TST but less INFγ positive results, thus reflecting the heterogeneity of this group and underscoring the importance of using clear reporting group categories based on the certainty of diagnosis to facilitate the interpretation of data.

Our findings therefore demonstrate that both INFγ and IP10 identify children with latent and active TB. IP10 is less affected by the presence of HIV co-infection than INFγ and has the potential to increase the sensitivity of the IGRAS when used in combination with INFγ. INFγ, IP10 and TST however are unable to differentiate between latent and active disease.

Longitudinal studies to describe the natural variation of these markers over time and their ability to identify children with latent infections at risk of disease progression or children at risk of poor treatment response after initiation of anti-TB therapy are needed. However, given the high cost of the IGRAS and the high proportion of indeterminate results obtained, control programmes should refrain from incorporating these tests until clear advantages over the TST are demonstrated.
